# Non-Linear Interactions Determine the Impact of Sea-Level Rise on Estuarine Benthic Biodiversity and Ecosystem Processes

**DOI:** 10.1371/journal.pone.0068160

**Published:** 2013-07-08

**Authors:** Tsuyuko Yamanaka, David Raffaelli, Piran C. L. White

**Affiliations:** 1 Environment Department, University of York, York, United Kingdom; 2 School of Environmental Sciences, University of Liverpool, Liverpool, United Kingdom; University College Dublin, Ireland

## Abstract

Sea-level rise induced by climate change may have significant impacts on the ecosystem functions and ecosystem services provided by intertidal sediment ecosystems. Accelerated sea-level rise is expected to lead to steeper beach slopes, coarser particle sizes and increased wave exposure, with consequent impacts on intertidal ecosystems. We examined the relationships between abundance, biomass, and community metabolism of benthic fauna with beach slope, particle size and exposure, using samples across a range of conditions from three different locations in the UK, to determine the significance of sediment particle size beach slope and wave exposure in affecting benthic fauna and ecosystem function in different ecological contexts. Our results show that abundance, biomass and oxygen consumption of intertidal macrofauna and meiofauna are affected significantly by interactions among sediment particle size, beach slope and wave exposure. For macrofauna on less sloping beaches, the effect of these physical constraints is mediated by the local context, although for meiofauna and for macrofauna on intermediate and steeper beaches, the effects of physical constraints dominate. Steeper beach slopes, coarser particle sizes and increased wave exposure generally result in decreases in abundance, biomass and oxygen consumption, but these relationships are complex and non-linear. Sea-level rise is likely to lead to changes in ecosystem structure with generally negative impacts on ecosystem functions and ecosystem services. However, the impacts of sea-level rise will also be affected by local ecological context, especially for less sloping beaches.

## Introduction

Climate change is expected to have significant effects on the marine environment through temperature change, ocean acidification, and accelerated sea-level rise, due to thermal expansion and melting of ice sheets [1]. Warmer sea temperatures are likely to cause changes in abundance, diversity and size composition of zooplankton [2], [3], increases in abundance of southern invertebrate species and decreases of northern species in the northern hemisphere [4]. Acidification may have both direct and indirect impacts, via changes in the phytoplankton community, on bacteria and zooplankton [5], [6], [7]. Some of the greatest impacts of sea-level rise are likely to occur in intertidal sediment habitats. Intertidal habitats will rarely be allowed to transgress inland due to the high value of real estate and land behind the existing high tide zone, so they will be squeezed between rising sea-level and hard coastal defences. Coastal squeeze leads to the loss of intertidal area, sedimentary shifts towards coarser particles and more reflective morphodynamic states, greater tidal velocity, changes in water depth and (for estuaries) salinity, and increased storm surges [1], [8], [9], [10].

Estuarine soft sediment habitats and their associated biodiversity are of great functional importance for the entire marine ecosystem and for human wellbeing in terms of the ecosystem services they produce, including high primary productivity, high secondary productivity, nutrient cycling, climate regulation, pollution control, decomposition, biodegradation and recreation [8], [11], [12], [13], [14], [15], [16], [17]. They also provide nursery grounds for marine fish, and feeding and breeding areas for migratory birds and other species [18]. Many of these services are underpinned by ecosystem processes occurring within the benthos [19], [20], [21]. Understanding the likely impacts of the physical constraints imposed by sea level rise on benthic assemblages is therefore important for evaluating and managing threats to future ecosystem service provision.

Coarser sediment size, steeper beach slopes and higher exposure to wave action are predicted for intertidal areas of the typical V-shaped estuaries that characterise much of the European coastline [9], [22]. Changes in these physical beach characteristics are likely to have an impact on benthic invertebrate biomass and body size distribution [22], [23], [24], and consequently on ecosystem processes. However, the nature of these impacts is likely to be determined by the interactions among the physical constraints of particle size, beach slope and exposure, collectively known as beach morphodynamic state [23], [25], rather than any of these factors acting alone. At one end of the morphodynamic range, dissipative beaches with finer particles and gentle slopes have been shown to support a higher number of species and a more abundant macrofauna [26], [27], [28], [29], whilst the opposite is true for reflective beaches at the other end of the range. Meiofauna are likely to be less affected than macrofauna by changes in exposure and sediment particle size [30].

Here we extend previous considerations of the potential impacts on estuarine benthic organisms of physical changes associated with sea-level rise to the ecosystem processes with which they are involved. We examine the relationships between abundance, biomass, and community metabolism of benthic organisms with beach slope, particle size and exposure, using samples that span a range of conditions from three very different locations in the UK. We test the following specific hypotheses [26], [27], [28]: (1) Total invertebrate abundance, biomass, and species richness will decline in response to increases in average sediment grain, steeper beach profiles and greater exposure to wave action; and (2) these changes will also be reflected in changes in community metabolism, a measure of ecosystem functioning. We also specifically consider the interactions between the different physical constraints and local ecological context in determining the overall impacts on beach fauna.

## Methods

### Ethics Statement

No specific permits were required for the field studies. All the sites sampled are public beaches, except for the Ythan which is a National Nature Reserve. Samples were taken from the Ythan under the permit issued by Scottish Natural Heritage to OceanLab, University of Aberdeen (Dr Martin Solan). None of the field studies involved endangered or protected species.

### Study Sites and Sampling Method

**Figure 1:**
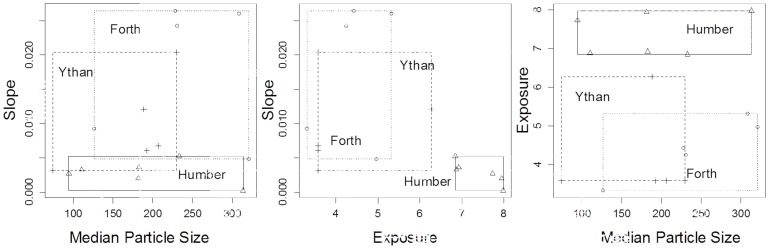
The range of median particle size, slope and exposure for each estuary.

We chose three estuaries within the UK: the Humber (from 53°33′20″ to 53°34′50″ N, and from 0°00′40″ to 0°03′20″ W), the Ythan (from 57°18′50″ to 57°20′20″ N, and from 1°59′20″ to 2°01′ 10″ W) and the Firth of Forth/the Forth estuary (from 55°57′30″ to 56°00′30″ N, and from 3°06′30″ to 3°31′00″ W) on the basis of their wide geographical spread within the UK and previous history of research. We selected five or six sampling stations on each of these estuaries to provide a range of particle sizes, slopes and exposures (Fig. 1). The sampling stations were restricted to a defined section of the outer (marine) salinity gradient (>30, spring high tide) to minimise any potentially confounding effects of salinity [31].

At the mid-tide level at each sampling station, to correspond with maximum invertebrate abundance, a cylindrical core (10.4 cm diameter) was pushed into the sediment to the depth of 10 cm on a randomly chosen surface to sample macrofauna, meiofauna and sediment, thus ensuring that all variables were measured at a similar scale (grain). Sampling stations were established using a map and previous surveys [31], [32], [33] Four widely-spaced replicates were taken for macrofauna, one sample for meiofauna and one sample for sediment. No replicates were taken for meiofauna as the sampling unit (86 cm^2^) is far greater than the scale of patchiness for this group (c. 3–10 cm^2^) [34], such that variance between samples of this size is extremely low.

### Biological Measurements

Macrofauna were separated from sediment using a 500 µm mesh, preserved in 70% ethanol, identified to species level wherever possible, and counted, using a low-power microscope. Meiofauna were separated from sediment using a 64 µm mesh, preserved in 70% ethanol and stained with Rose Bengal (5 mg l^−1^ 70% ethanol). Because of the large core size used for sampling meiofauna and the large number of individuals collected, meiofauna from each site were sub-sampled after extraction and homogenisation. Meiofauna in these sub-samples ( = 1/200^th^ of core) for each site were identified to the lowest possible taxon, counted and measured to provide body dimensions for the calculation of body mass (Table S1).

#### Calculation of body mass

Body dimensions of individual animals were measured under a high-power microscope, and converted to body mass (dry weight) using established relationships ([35]; Table S1). Where densities were high, body dimensions were measured and body mass calculated for a sub-sample, and then scaled up to the number in the full sample.

#### Community metabolism

Community metabolism was calculated as oxygen consumption by all individual invertebrates using data on mass-specific oxygen consumption factors derived by Banse [36], as used by Gerlach, Hahn, et al. [37]. Abundances of macrofauna and meiofauna were expressed as numbers core^−1^, biomass as Ash Free Dry Weight (AFDW) as mg core^−1^, and oxygen consumption as µlO_2_ hr^−1^ core^−1^. Thus,

Log_10_ (Oxygen Consumption) = −0.2599*Log_10_ (AFDW) + 0.51 for macrofauna;

Log_10_ (Oxygen Consumption) = −0.24*Log_10_ (AFDW) + 0.0096 for meiofauna; and

Log_10_ (Oxygen Consumption) = −0.2605*Log_10_ (AFDW) − 0.4424 for foraminifera [34], [35].

### Beach Physical Characteristics

#### Sediment particle size

Sediment cores were oven-dried at 80°C for at least 24 hours, after which the sample was thoroughly homogenised. Particle size composition was determined by dry sieving the material through a tower of sieves of mesh 4.75 mm, 2.8 mm, 1 mm, 500 µm, 250 µm, 125 µm and 64 µm. Median particle size (µm), 25% and 75% quartiles (*Q*_25_, *Q*_75_ in µm) and the sorting coefficient *QDφ
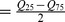
* were determined by standard probit analysis. Silt content was taken as the weight of material passing through a 64 µm mesh by wet sieving.

#### Beach slope

The slope at each sampling station was calculated as:
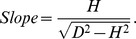
where *H* = vertical height (m) difference between the low and high shore, and *D* = distance (m) down the beach between the low and high points. Height difference was measured using a dumpy level and a staff, and distance using a surveyor’s tape. Because the values for slope on these beaches are very small, they are presented in the text as slope×10^3^, to facilitate inter-site comparisons.

#### Exposure

Exposure was calculated using a modified form of the Thomas Exposure Index (TEI) [38], derived from wind velocity, direction, duration and the effective fetch:
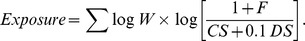
where *W* = (percentage of time wind blows in a sector (22.5 degree sectors of the compass rose)/100) × (mean wind speed (kn)^2^).

*F* = Fetch in nautical miles (100 nautical miles maximum).

*CS* = Extent in nautical miles of water <6 m deep adjoining the shore.

*DS* = Extent in nautical miles of water within the fetch <6 m deep, but not adjoining the shore.

*W* was calculated using the wind data from windfinder.com, and *F*, *CS*, and *DS* were derived from UK Admiralty charts.

### Statistical Analysis

Statistical models (mixed model for macrofauna and linear regression with GLS extension for meiofauna) were developed [39], [40] for abundance, biomass and oxygen consumption of macrofauna and meiofauna, in relation to the physical beach variables. Physical beach variables (median particle size, beach slope and wave exposure) were measured for each station, and four replicates were included in the model for macrofaunal abundance, biomass and oxygen consumption and no replicates were included for meiofaunal abundance, biomass and oxygen consumption. Silt content was not included in the models as it was strongly correlated with median particle size, and the sorting coefficient QDφ was not significant in any models. We included the three estuaries initially as a random local ecological effect in all models; this term was highly significant in the model for macrofauna, although not in that for meiofauna. For the macrofauna model, we therefore used a mixed modelling approach, including median particle size, beach slope and exposure as physical fixed effects and estuary as a random local effect. For the meiofauna model, we included median particle size, beach slope and exposure as effects, and used a linear regression model with a GLS extension to allow for unequal variance associated with median particle size [41], [42]. All the possible two and three way interactions were included in both macrofaunal and meiofaunal models.

## Results

### Beach Physical and Biological Characteristics

The Humber and the Forth estuaries had a similar range of particle size (with the Ythan range slightly lower), and the Humber had much higher exposure than the Ythan and the Forth (no overlap in range). The Ythan and the Forth also had a similar range of beach slopes, whereas those for the Humber were much shallower (Table 1, Fig. 1).

**Table 1:** Summary of physical characteristics of each estuary showing the median values (and ranges) encountered within each estuary.

	Estuary		
	Humber	Ythan	Forth
Median particle size (µm)	183 (95–314)	192 (74–230)	230 (126–321)
Silt content (%)	3.8 (2–54.8)	20.4 (12.4–67.6)	1.0 (0.6–22.6)
Sorting coefficient QDφ	44 (29–154)	81 (40–133)	53.5 (49–73)
Slope (×10^3^)	2.9 (0.2–5.2)	6.8 (3.1–20.4)	16.8 (4.8–26.5)
Exposure	7.33 (6.84–7.98)	3.57 (3.57–6.27)	4.34 (3.32–5.32)

Exposure was calculated as a modified Thomas Exposure Index (see text).

The Ythan and the Humber had similar numerically dominant species and species richness of macrofauna, but differed markedly in the numbers of individuals recorded (Table 2). The Forth had intermediate abundances, but a greater range of species, and was dominated numerically by polychaetes. The Forth had the most meiofaunal taxa represented, including mites (Acarina) and archiannelids. Nematodes and foraminiferans (live as opposed to dead shells) occurred in large numbers, especially on the Humber and the Ythan, and Copepods and Turbellarians were abundant on the Ythan.

**Table 2:** Summary of biological characteristics of each estuary showing the median values (and ranges) encountered within each estuary.

	Estuary		
	Humber	Ythan	Forth
Macrofauna			
* Hydrobia ulvae*	8.75 (0–87.5)	48 (0.25–380.5)	0.75 (0–36)
* Macoma balthica*	2.50 (0.5–42.25)	0.75 (0.25–7.5)	0 (0–4.25)
* Pygospio elegans*	1.00 (0.25–6.25)	42.5 (1.5–92.25)	1.75 (0.75–170.75)
* Cerastoderma edule*	0.75 (0.25–1.25)	0 (0–0.75)	0.25 (0–3.5)
* Etone longa*	0 (0–0.75)	1 (0–0.75)	0.5 (0–3)
* Nereis diversicolor*	0 (0–0.25)	2.25 (0.25–8.25)	0.25 (0–2)
* Corophium volutator*	0 (0–0.25)	167.25 (8.25–468.25)	0 (0–0.25)
Midge fly larvae	0 (0–0.25)	0.25 (0–0.75)	0 (0–0)
Collembola	0 (0–1)	0 (0–1.75)	0 (0–0)
Capitellidae	0 (0–1.25)	0 (0–0)	1.75 (0.5–403)
Syllidae	0.375 (0–1.75)	0 (0–0)	0 (0–0)
* Scoloplos armiger*	0.125 (0–0.75)	0 (0–0)	0 (0–0)
* Urothoe brevicornis*	0 (0–0.25)	0 (0–0)	0.25 (0–0.5)
* Arenicola marina*	0 (0–0.25)	0 (0–0)	0 (0–0.25)
Cumacea	0 (0–0.25)	0 (0–0)	0 (0–0.75)
Other Oligochaetes	1.375 (0–17.5)	0 (0–0)	1.5 (0–30)
Nephtys caeca	1.25 (0–2.25)	0 (0–0)	0 (0–0)
* Mytilus edulis*	0 (0–0)	0 (0–0.5)	2 (0–7.25)
* Retusa abtusa*	0 (0–0)	0 (0–0.5)	0 (0–5)
* Tubifex costatus*	0 (0–0)	30.25 (9.75–132.5)	0 (0–0)
* Tubificoides benedeni*	0 (0–0)	47.75 (1.75–64)	4.25 (0.25–104)
* Manayunkia aestuarina*	0 (0–0)	11 (05–84.5)	0 (0–1)
* Carcinus menas*	0 (0–0)	0 (0–0.25)	0 (0–0)
* Tetrastemma melanocephalum*	0 (0–0)	0 (0–0.25)	0 (0–0)
* Streblospio benedicti*	0 (0–0)	0 (0–0)	9.75 (1.25–50)
* Littorina littorea*	0 (0–0)	0 (0–0)	0 (0–0.25)
Meiofauna			
Forminiferans	25369 (3103–111498)	48223 (24896–187471)	4001 (308–59000)
Nematodes	16800 (4673–34669)	19974 (14410–58924)	4212 (2916–13235)
Oligochaetes	571 (178–2140)	571 (143–1855)	778 (454–3256)
Copepods	375 (36–892)	17121 (6335–47253)	243 (178–8181)
Turbellarians	0 (0–0)	25681 (12983–112140)	1231 (405–5054)
Ostracods	0 (0–0)	0 (0–0)	259 (0–4666)
Archiannerids	0 (0–0)	0 (0–0)	194 (0–3094)
Acarina	0 (0–0)	0 (0–0)	0 (0–97)

Values for macrofauna and meiofauna are averages per core.

### Relationships of Macro- and Meiofauna with Beach Physical Characteristics

#### Macrofaunal abundance, biomass and oxygen consumption

**Figure 2:**
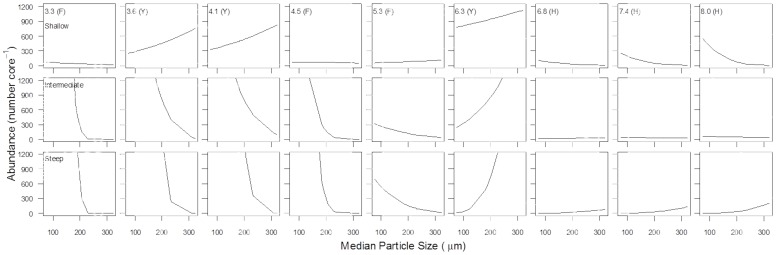
Predicted macrofaunal abundance based on the minimal adequate regression model for each estuary. The values on graphs show the predictions for minimum, mean and maximum exposure values of each estuary. H, Y, and F represent the Humber, the Ythan, and the Forth, respectively. Shallow, Intermediate, and Steep represent the minimum (top row), mean (middle row) and maximum (bottom row) slope of each estuary. Minimal adequate model is the model that contains the minimum number of predictors which are chosen here by stepwise multiple regression.

Median particle size and beach slope had a similarly strong influence on the abundance of macrofauna (Table 3), whilst exposure had relatively less influence on abundance. The three-way interaction term particle size × beach slope × exposure was also significant. Sediment particle size, beach slope and wave exposure are usually associated to each other (the beach with high wave exposure tend to have steeper beach profile and coarser sediment). Because the local ecological effect (estuary) was highly significant, the graphs to show the predictions of the mixed model for macrofaunal abundance were made for minimum, mean and maximum exposure and beach slope for each estuary separately (Fig. 2).

**Table 3:** L-ratios for regression models of abundance, biomass and oxygen consumption for macrofauna and meiofauna with respect to the single factors median particle size, slope and exposure (each *df* = 4) and the three-way interaction between them (*df* = 1).

	Median particle size	Slope	Exposure	Particle size×slope×exposure
Macrofauna				
Abundance	42.97***	42.91***	34.97***	27.76***
Biomass	24.38**	36.61***	22.82**	8.7*
O_2_ consumption	28.01***	37.31***	25.1***	13.85**
Meiofauna				
Abundance	15.36*	15.8*	14.59*	6.74*
Biomass	15.38*	27.63***	30.51***	8.27*
O_2_ consumption	20.45**	24.91**	25.4***	11.78**

*** *P*<0.0001, **P<0.001 and *P<0.01.

For shallow-sloping beaches, there were no consistent relationships between abundance and exposure or median particle size across the estuaries, and the local ecological effect dominated the patterns observed (Fig. 2). Thus, for shallow-sloping beaches, there was a weak negative relationship between abundance and particle size for the Humber stations, a slightly stronger, positive one for all the Ythan stations and no relationship for the Forth stations. However, for intermediate and steep beaches, there were clear and consistent non-linear relationships between abundance and exposure and particle size, which overrode the local ecological effect (Fig. 2). For the most sheltered beaches, there were strong negative relationships between abundance and particle size. However, as exposure increased, this relationship became more weakly negative and then positive at intermediate exposures, and then became negligible or very weakly positive at the highest exposures (Fig. 2).

Macrofaunal biomass and oxygen consumption exhibited the same non-linear relationships with physical factors as macrofaunal abundance (Figs. S1 and S2). The most significant single factor affecting both biomass and oxygen consumption was beach slope (Table 3), followed by median particle size and exposure. The three-way interaction term particle size × beach slope × exposure was also significant for both macrofaunal biomass and oxygen consumption (Table 3). Biomass and oxygen consumption show contrasting trends because weight-specific oxygen consumption increases as organisms become smaller [36], [37].

#### Meiofaunal abundance, biomass and oxygen consumption

**Figure 3:**
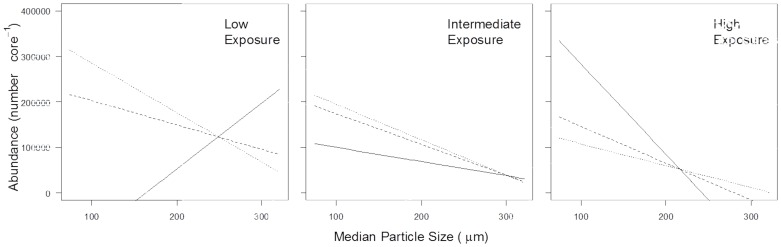
Predicted meiofaunal abundance for low, intermediate and high exposure, based on minimal adequate regression model. The lines in the panels represent steep slope (solid line), intermediate slope (dashed line) and shallow slope (dotted line).

Beach slope had the greatest effect on meiofaunal abundance (Table 3), followed by median particle size and exposure. The three-way interaction term particle size × beach slope × exposure also had a significant effect on meiofaunal abundance; meiofaunal abundance decreased with increasing exposure and increasing particle size on the shallow- and intermediate-slope beaches (Fig. 3). However, on the steep beaches, there was evidence of a non-linear response to the interaction between exposure and particle size, similar to that observed for macrofauna.

Meiofaunal biomass and oxygen consumption exhibited the same non-linear relationships with physical factors as meiofaunal abundance (Figs. S3 and S4). Meiofaunal biomass and oxygen consumption were affected most significantly by exposure, followed by beach slope and median particle size (Table 3). The three-way interaction term particle size × beach slope × exposure was a significant factor determining both meiofaunal biomass and oxygen consumption. The two-way interactions were also significant for all the models, but these lower term interactions are not discussed here.

## Discussion

Our analyses revealed strong relationships between macrofaunal and meiofaunal abundance, biomass and oxygen consumption and beach slope, particle size and exposure. That relationships exist is not unexpected, but here we have been able to tease out the relative importance of the different variables and, more importantly, their linear or non-linear nature. The most significant factor affecting macrofauna was slope, followed by particle size and exposure. For meiofauna, the most significant factor was exposure, followed by slope and particle size. For the shallowest beaches, the local context (which estuary it was) overrode these other relationships, but for intermediate and steeper beaches, the physical factors were the dominant influences. The overall pattern for the relationship for intermediate and steep beaches was negative, with invertebrate abundance, biomass and oxygen consumption declining with increasing slope, particle size and exposure, but the effects were complex and non-linear, with the relationship switching at intermediate levels of exposure.

Previous studies have shown that dissipative beaches with fine particles and gentle slopes support higher numbers of species, in greater abundance and biomass [15], [23], [26], [27], [28]. The present study confirms these trends for the meiofauna, showing declines in abundance, biomass, and oxygen consumption with increasing particle size, beach slope, and exposure. However, our results go much further than previous analyses in demonstrating that under certain conditions these relationships are likely to be more complex and non-linear. This has important implications for the impact of sea-level rise on ecosystem service provision such as reduced food resources, reduced water quality, reduced primary productivity and change in release of nutrient to the overlying water column, since the effects of steeper slopes, increasing particle size and increased exposure on ecosystem structure and function will be moderated by local conditions, especially for less sloping beaches.

McLachlan et al. [30] reported greater numbers of meiofaunal harpacticoid copepods and fewer nematodes in relation to larger median particle sizes. Those authors found that nematodes tended to dominate in finer sands (<330 µm), with overall biomass higher, similar to the present study, where all sites had particle sizes <330 µm and were also dominated by nematodes. In contrast, Rodriguez et al. [43] found increased meiofaunal biomass with increasing wave exposure, but their sites were much more exposed. Taken together, these results emphasise the complexity of the relationships between biodiversity elements and physical constraints on beaches, which probably contribute to the non-linear trends observed by others for ecosystem service provision in coastal ecosystems [14].

### Interactions between Beach Physical Factors and Local Ecological Context

McLachlan [25] and Raffaelli and Hawkins [23] argued that the interaction between particle size, beach slope and exposure is probably as important, if not more so, for predicting changes in beach fauna than the effect of any single physical factor. This is formally confirmed in the present study for the first time: the simple bivariate relationships depicted in the literature such as graphs of abundance or species richness against sediment particle size (e.g. [23], p59, Fig. 2.10) are clearly inadequate descriptions of the true, more complex relationships, and would be misleading if used to predict impacts of sea-level rise.

The meiofaunal models revealed linear relationships between variables, but all the macrofaunal models indicate non-linear relationships. Since maximum faunal abundance and diversity may change its exact location along the intertidal gradient depending on exposure, and the sampling stations were fixed at a geographical mid-tide level in this study, it could be argued that the observed non-linear responses of macrofauna with exposure-slope could be due to sampling at this fixed point. However, we think this is unlikely given that the meiofaunal relations might then have been expected to be similarly non-linear. The underlying causes of the non-linear macrofaunal relationships thus remain unclear.

The location of the three estuaries was highly significant for models of abundance, biomass and oxygen consumption of macrofauna, but not for the meiofauna. This suggests that the importance of physical factors outweighs that of local context for meiofauna, although for macrofauna, this is only the case under the limited conditions of less sloping beaches. The exact nature of these local effects is unknown, but they must reflect other local variables which we did not measure, such as differences in predation by birds and fish, or differences in water quality.

### Impact of Sea-level Rise on Ecosystem Services from Estuarine Systems

Accelerated sea-level rise is expected to make beaches coarser and steeper, and more reflective in morphodynamic state [10], [29]. The predicted increase in the frequency of storm surges [1] will add to this effect. Our results suggest that if beach physical factors change as predicted, then sea-level rise will make estuarine inter-tidal areas less diverse and less productive, through declines in abundance, biomass, and community metabolism as well as through the loss of area due to coastal squeeze. Mechanistic understanding of the relationships between biodiversity, ecosystem functions and ecosystem services remains uncertain for estuaries [42], although experimental work on benthic biodiversity and ecosystem functioning suggests that reductions in macro- and meiofaunal biomass and metabolism are likely to have significant effects in reducing nutrient cycling within sediments and release of nutrients to the overlying water column [19], [39], [44], [45], [46]. Our results have highlighted the complex and non-linear nature of these relationships, and more precise prediction of the impact of sea-level rise on ecosystem service provision will depend on a much improved understanding of the interactive effects of local ecosystem contexts and globally-operating physical constraints on ecosystem structure and function.

## Supporting Information

Figure S1 **Predicted macrofaunal biomass based on the minimal adequate regression model for each estuary.** The values on graphs show minimum, mean, maximum exposure values of each estuary. H, Y, and F stand for the Humber, the Ythan, and the Forth, respectively. Shallow, Intermediate, and Steep represent the minimum (top row), mean (middle row) and maximum (bottom row) slope of each estuary.
(TIF)

Figure S2 **Predicted macrofaunal oxygen consumption based on the minimal adequate regression model for each estuary.** The values on graphs show minimum, mean, maximum exposure values of each estuary. H, Y, and F stand for the Humber, the Ythan, and the Forth, respectively. Shallow, Intermediate, and Steep represent the minimum (top row), mean (middle row) and maximum (bottom row) slope of each estuary.
(TIF)

Figure S3 **Predicted meiofaunal biomass for low, intermediate and high exposure, based on minimal adequate regression model.** The lines in the panels represent steep slope (solid line), intermediate slope (dashed line) and shallow slope (dotted line).
(TIF)

Figure S4 **Predicted meiofaunal oxygen consumption for low, intermediate and high exposure, based on minimal adequate regression model.** The lines in the panels represent steep slope (solid line), intermediate slope (dashed line) and shallow slope (dotted line).
(TIF)

Table S1 **Relationships between morphological dimensions and body weight.**
(DOCX)

## References

[1] IPCC Working Group 1 (2007) Observations: Oceanic Climate Change and Sea Level. In: Solomon S, Qin D, Manning M, Marquis M, Averyt K et al.., editors. Climate Change 2007 The Physical Science Basis. Cambridge: Cambridge University Press.

[2] Richardson AJ (2008) In hot water: zooplankton and climate change. ICES Journal of Marine Science: Journal du Conseil 65: 279–295.

[3] Roemmich D, McGowan J (1995) Climatic warming and the decline of zooplankton in the California current. Science 267: 1324–1326. 17812604 10.1126/science.267.5202.1324

[4] Barry JP, Baxter CH, Sagarin RD, Gilman SE (1995) Climate-related, long-term faunal changes in a California rocky intertidal community. Science 267: 672–675. 17745845 10.1126/science.267.5198.672

[5] Hofmann GE, Barry JP, Edmunds PJ, Gates RD, Hutchins DA, et al (2010) The Effect of Ocean Acidification on Calcifying Organisms in Marine Ecosystems: An Organism-to-Ecosystem Perspective. Annual Review of Ecology, Evolution, and Systematics 41: 127–147.

[6] Kurihara H, Shimode S, Shirayama Y (2004) Sub-lethal effects of elevated concentration of CO2 on planktonic copepods and sea urchins. Journal of Oceanography 60: 743–750.

[7] Lohbeck KT, Riebesell U, Reusch TBH (2012) Adaptive evolution of a key phytoplankton species to ocean acidification. Nature Geosci 5: 346–351.

[8] Fujii T, Raffaelli D (2008) Sea-level rise, expected environmental changes, and responses of intertidal benthic macrofauna in the Humber estuary, UK. Marine Ecology Progress Series 371: 23–35.

[9] Goss-Custard JD, McGrorty S, Kirby R (1990) Inshore birds of the soft coasts and sea-level rise. In: Beukema JJ, Wolff WJ, Brouns JJWM, editors. Expected Effects of Climate Change on Marine Coastal Ecosystem. Dordrecht: Kluwer Academic Publishers. 189–193.

[10] Pethick J (1993) Shoreline adjustments and coastal management: physical and biological processes under accelerated sea-level rise The Geographical Journal. 159: 162–168.

[11] Balvanera P, Pfisterer AB, Buchmann N, He J-S, Nakashizuka T, et al (2006) Quantifying the evidence for biodiversity effects on ecosystem functioning and services. Ecology Letters 9: 1146–1156. 16972878 10.1111/j.1461-0248.2006.00963.x

[12] Hooper DU, Adair EC, Cardinale BJ, Byrnes JEK, Hungate BA, et al (2012) A global synthesis reveals biodiversity loss as a major driver of ecosystem change. Nature 486: 105–108. 22678289 10.1038/nature11118

[13] Kennish MJ (2002) Environmental threats and environmental future of estuaries. Environmental Conservetion 29: 78–107.

[14] Koch EW, Barbier EB, Silliman BR, Reed DJ, Perillo GM, et al (2009) Non-linearity in ecosystem services: temporal and spatial variability in coastal protection. Frontiers in Ecology and the Environment 7: 29–37.

[15] McLusky DS, Elliot M (2004) The Estuarine Ecosystem Oxford: Oxford University Press. 214 p.

[16] Palumbi SR, Sandifer PA, Allan JD, Beck MW, Fautin DG, et al (2009) Managing for ocean biodiversity to sustain marine ecosystem services. Frontiers in Ecology and the Environment 7: 204–211.

[17] White PCL, Godbold JA, Solan M, Wiegand J, Holt AR (2010) Ecosystem services and policy: a review of coastal wetland ecosystem services and an efficiency-based framework for implementing the ecosystem approach. Issues in Environmental Science and Technology In press.

[18] Galbraith H, Jones R, Park R, Clough J, Herrod-Julius S, et al (2002) Global Climate Change and Sea Level Rise: Potential Losses of Intertidal Habitat for Shorebirds. Waterbirds: The International Journal of Waterbird Biology 25: 173–183.

[19] Bulling MT, White PCL, Raffaelli D, Pierce GJ (2006) Using model systems to address the biodiversity - ecosystem functioning process. Marine Ecology Progress Series 311: 295–309.

[20] Ieno EN, Solan M, Batty P, Pierce GJ (2006) How biodiversity affects ecosystem functioning: roles of infaunal species richness, identity and density in the marine benthos. Marine Ecology Progress Series 311: 263–271.

[21] Raffaelli D (2006) Biodiversity and ecosystem functioning: issues of scale and trophic complexity. Marine Ecology Progress Series 311: 285–294.

[22] McLachlan A, Brown A (2006) The Ecology of Sandy Shores. London: Elsevier.

[23] Raffaelli D, Hawkins S (1996) Intertidal Ecology: Chapman&Hall.

[24] Yamanaka T, White PCL, Spencer M, Raffaelli D (2011) Patterns and processes in abundance–body size relationships for marine benthic invertebrates. Journal of Animal Ecology 81: 463–471. 22010834 10.1111/j.1365-2656.2011.01921.x

[25] McLachlan A (1990) Dissipative Beaches and Macrofauna Communitied on Exposed Intertidal Sands. Journal of Coastal Research 6: 57–71.

[26] Lercari D, Defeo O (2006) Large-scale diversity and abundance trends in sandy beach macrofauna along full gradients of salinity and morpodynamics. Estuarine Coastal and Shelf Science 68: 27–35.

[27] McLachlan A (1996) Physical factors in benthic ecology: effects of changing sand particle size on beach fauna. Marine Ecology Progress Series 131: 205–217.

[28] McLachlan A, Jaramillo E, Donn TE, Wessels F (1993) Sandy beach macrofauna communities and their control by the physical environment: a geographical comparison. Journal of Coastal Research 15: 27–38.

[29] Yamanaka T, Raffaelli D, White PCL (2010) Physical determinants of intertidal communities on dissipative beaches: Implications of sea-level rise. Estuarine, Coastal and Shelf Science 88: 267–278.

[30] McLachlan A, Wooldridge T, Dye AH (1981) The ecology of sandy beaches in Southern Africa. South African Journal of Zoology 16: 219–231.

[31] Fujii T (2007) Spatial patterns of benthic macrofauna in relation to environmental variables in an intertidal habitat in the Humber estuary, UK: Developing a tool for estuarine shoreline management. Estuarine Coastal and Shelf Science 75: 101–119.

[32] Bolam SG, Fernandes TF, Huxham M (2002) Diversity, Biomass, and Ecosystem Processes in the Marine Benthos. Ecological Monographs 72(4): 599–615.

[33] Leach JH (1970) Epibenthic Algal Production in an Intertidal Mudflat. Limnology and Oceanography 15: 514–521.

[34] Hall SJ, Raffaelli D, Thrush SF (1994) Patchiness and disturbance in shallow water benthic assemblages. *British Ecological Society Symposium, April 1992*, Aquatic Ecology: scale, pattern and process. Oxford: Blackwells. 333–375.

[35] Feller RJ, Warwick RM (1988) Enegetics. In: Higgins RP, Thiel H, editors. Introduction to the Study of Meiofauna. London: Smithsonian Institution Press. 181–196.

[36] Banse K (1982) Mass-Scaled Rates of Respiration and Intrinsic Growth in Very Small Invertebrates. Marine Ecology 9: 281–297.

[37] Gerlach SA, Hahn AE, Schrage M (1985) Size spectra of benthic biomass and metabolism. Marine Ecology 26: 161–173.

[38] Thomas MLH (1986) A physically derived exposure index for marine shorelines. Ophelia 25: 1–13.

[39] Bulling MT, Solan M, Dyson KE, Hernandez-Milian G, Luque P, et al (2008) Species effects on ecosystem processes are modified by faunal responses to habitat composition. Oecologia 158: 511–520. 18836748 10.1007/s00442-008-1160-5

[40] Zuur AF, Ieno EN, Smith GM (2007) Analysing ecological data. New York: Springer.

[41] Pinheiro J, Bates D, DebRoy S, Sarker DS (2006) nlme(3.1–90): an R package for fitting and comparing Gaussian linear and nonlinear mixed-effects models.

[42] Pinheiro JC, Bates DM (2000) Mixed-effects models in S and S-plus. New York: Springer.

[43] Rodriguez GJ, Mariano L, Lopez J (2002) Meiofauna distribution along a gradient of sandy beaches in northern Spain. Estuarine Coastal and Shelf Science 58: 63–69.

[44] Solan M, Raffaelli DG, Paterson DM, White PCL, Pierce GJ (2006 ) Theme Section. Marine biodiversity and ecosystem function: empirical approaches and future research needs. Introduction.. Marine Ecology Progress Series 311: 175–178.

[45] Dyson KE, Bulling M, Solan M, Raffaelli D, White PCL, et al (2007) Influence of macrofaunal assemblages and environmental heterogeneity on microphytobenthic production in experimental systems. Proceedings of the Royal Society of London B 274: 2547–2554. 10.1098/rspb.2007.0922 PMC2275895 17698480

[46] Bulling MT, Hicks N, Murray L, Paterson DM, Raffaelli D, et al.. (2010) Marine biodiversity-ecosystem functions under uncertain environmental futures. Philosophical Transactions of the Royal Society of London B in press. 10.1098/rstb.2010.0022 PMC2880130 20513718

