# Supplementary figures and images for: Non-Linear Interactions Determine the Impact of Sea-Level Rise on Estuarine Benthic Biodiversity and Ecosystem Processes
Source: PLoS One. 2013 Jul 8;8(7):e68160. doi: 10.1371/journal.pone.0068160 (PMC3704648; doi:10.1371/journal.pone.0068160)

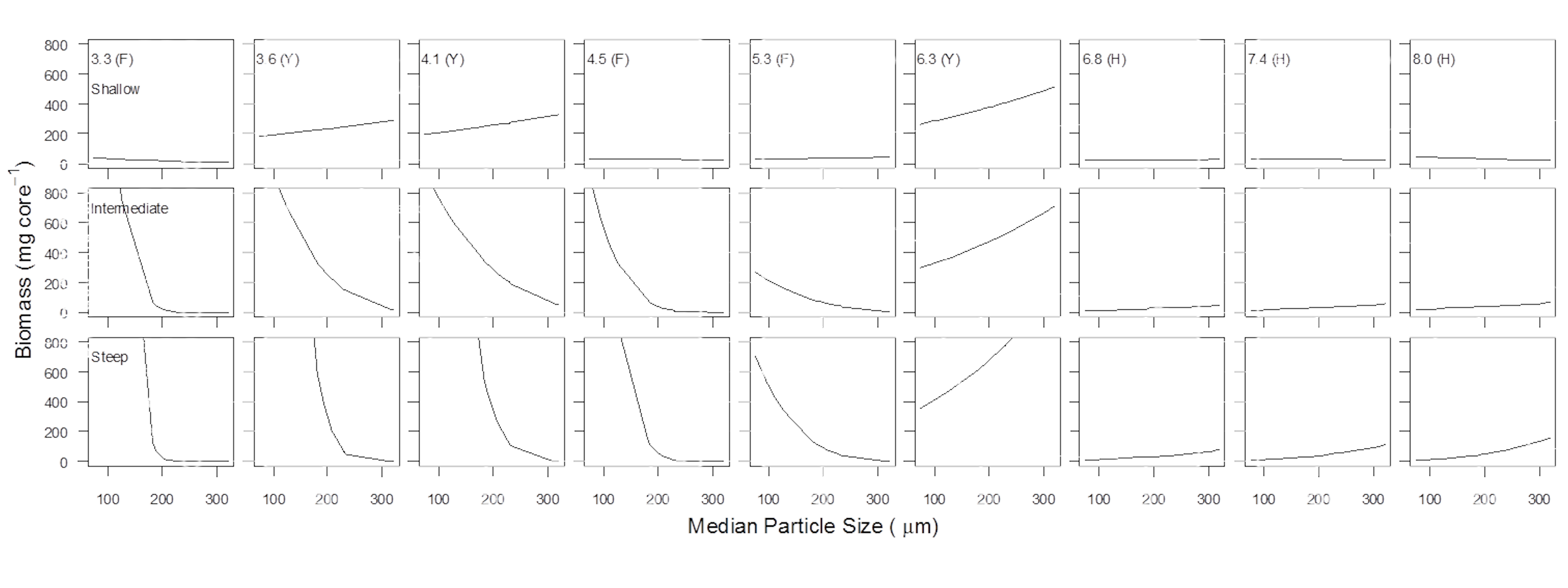

Supplement: Figure S1 — Predicted macrofaunal biomass based on the minimal adequate regression model for each estuary. The values on graphs show minimum, mean, maximum exposure values of each estuary. H, Y, and F stand for the Humber, the Ythan, and the Forth, respectively. Shallow, Intermediate, and Steep represent the minimum (top row), mean (middle row) and maximum (bottom row) slope of each estuary. (TIF) [file pone.0068160.s001.tif]

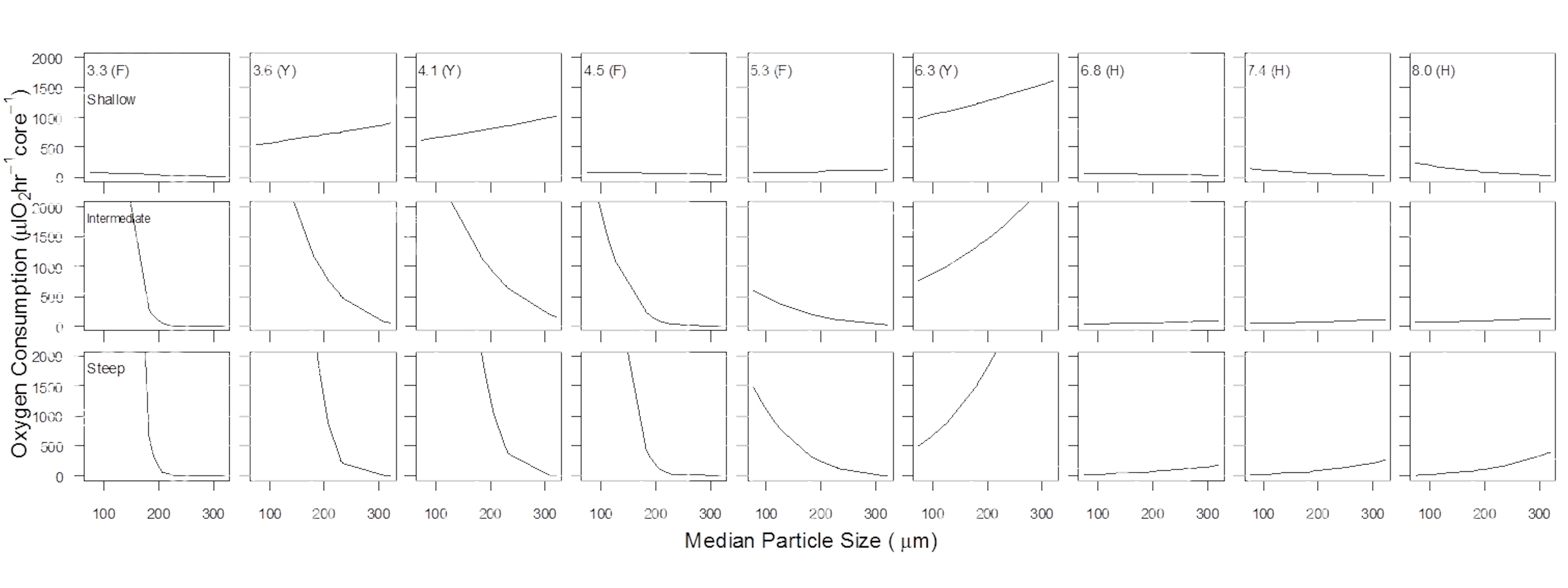

Supplement: Figure S2 — Predicted macrofaunal oxygen consumption based on the minimal adequate regression model for each estuary. The values on graphs show minimum, mean, maximum exposure values of each estuary. H, Y, and F stand for the Humber, the Ythan, and the Forth, respectively. Shallow, Intermediate, and Steep represent the minimum (top row), mean (middle row) and maximum (bottom row) slope of each estuary. (TIF) [file pone.0068160.s002.tif]

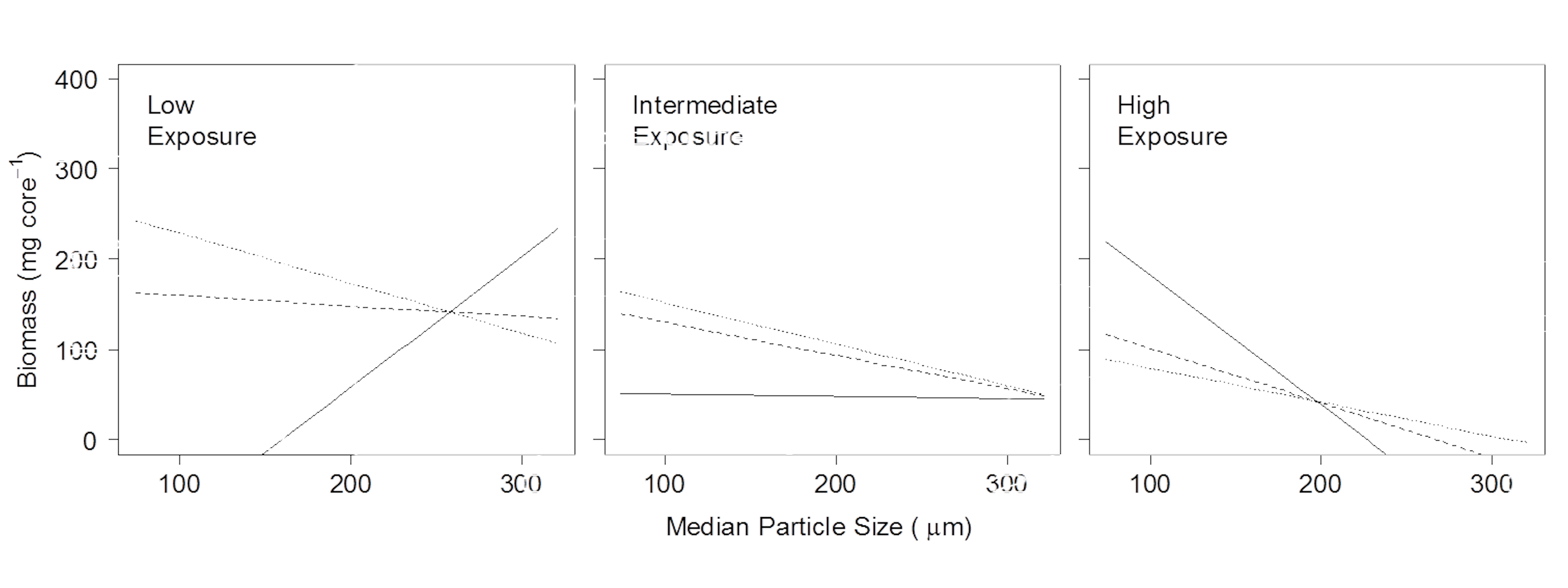

Supplement: Figure S3 — Predicted meiofaunal biomass for low, intermediate and high exposure, based on minimal adequate regression model. The lines in the panels represent steep slope (solid line), intermediate slope (dashed line) and shallow slope (dotted line). (TIF) [file pone.0068160.s003.tif]

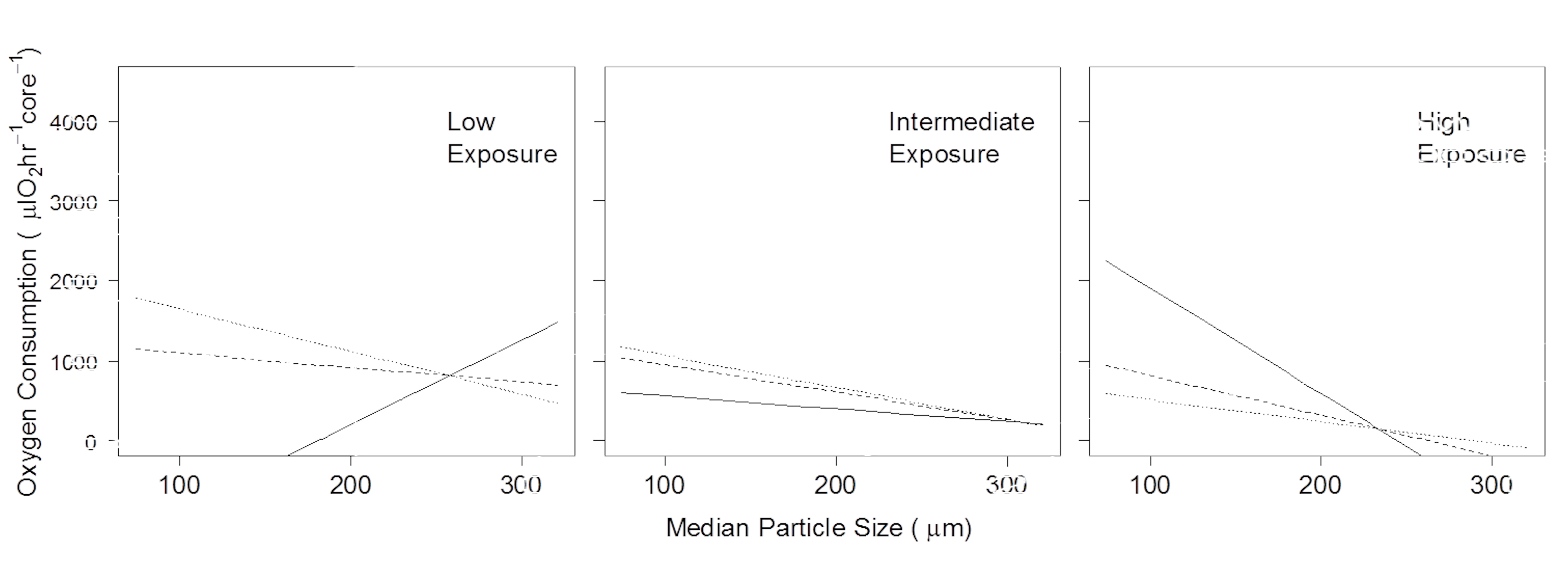

Supplement: Figure S4 — Predicted meiofaunal oxygen consumption for low, intermediate and high exposure, based on minimal adequate regression model. The lines in the panels represent steep slope (solid line), intermediate slope (dashed line) and shallow slope (dotted line). (TIF) [file pone.0068160.s004.tif]
