# Supplementary material for: Non-Linear Interactions Determine the Impact of Sea-Level Rise on Estuarine Benthic Biodiversity and Ecosystem Processes
Source: PLoS One. 2013 Jul 8;8(7):e68160. doi: 10.1371/journal.pone.0068160 (PMC3704648; doi:10.1371/journal.pone.0068160)
Supplement: Table S1 — Relationships between morphological dimensions and body weight. (DOCX) [file pone.0068160.s005.docx]

**Table S1.** Relationships between morphological dimensions and body weight

| Species | Relationships |
| --- | --- |
| **Macrofauna** |  |
| *Cerastoderma edule* | log_10_ (dry flesh in mg) = -1.9717 + 3.1084*log_10_ (body characteristic in mm)^a^ |
| *Corophium volutator* | log_10_ (dry flesh in mg) = -1.7088 + 2.4427*log_10_ (body characteristic in mm) ^a^ |
| *Hydrobia ulvae* | log_10_ (dry flesh in mg) = -1.5871 + 2.3631*log_10_ (body characteristic in mm) ^a^ |
| *Mytilus edulis* | log_10_ (dry flesh in mg) = -2.6887 + 3.215*log_10_ (body characteristic in mm) ^a^ |
| *Macoma balthica* | log_10_ (dry flesh in mg) = -1.4536 + 2.9414*log_10_ (body characteristic in mm) ^a^ |
| *Pygospio elegans* | Length = 1.69*Width (5^th^ setiger) + 1.56 ^b^ |
|  | V = L*W^2^*530 ^c^ |
|  | Dry weight (µg) = V*1.13*0.17 ^c, j^ |
| *Nereis(Hediste) diversicolor* | Wet weight (mg) = 25.65*Width (2^nd^ setiger) ^1.13 d^ |
| *Nephtys caeca* | Wet weight (mg) = 25.65*Width (2^nd^ setiger) ^1.13 d^ |
| *Eteone longa* | Wet weight (mg) = 25.65*Width (2^nd^ setiger) ^1.13 d^ |
| Oligochaeta | InDW = (InW_10_*1.62303) + (InL_10_*1.06101) – 1.90887^e^ |
| Collembola | W = 6.1894*L^3.119^*10^-9 f^ |
| *Carcinus menas* | W = 0.00025*CW^3.01 g^ |
| *Manayunkia aestuarina* | Log_10_ (Area) = -1.08381+1.92456*Log_10_ (Length) ^h^ |
|  | Volume = Area^2.23 i^ |
|  | Dry weight (µg) = V*1.13*0.25 ^c^ |
| *Retusa obtusa* | log_10_ (dry flesh in mg) = -1.5871 + 2.3631*log_10_ (body characteristic in mm) ^a^ |
| Capitellidae, Syllidae, | Length = 1.69*Width (5^th^ setiger) + 1.56 ^b^ |
| *Scoloplos armiger* | V = L*W^2^*530 ^c^ |
|  | Dry weight (µg) = V*1.13*0.17 ^c, j^ |
| Cumacea | V = L*W^2^*400 ^c^ |
|  | Dry weight (µg) = V*1.13*0.17 ^c, j^ |
| *Urothoe brevicornis* | V = L*W^2^*230 ^c^ |
|  | Dry weight (µg) = V*1.13*0.17 ^c, j^ |
| Meiofauna |  |
| Nematodes | V = L*W^2^*530, Dry weight (µg) = V*1.13*0.25 ^c^ |
| Ostracods | V = L*W^2^*450, Dry weight (µg) = V*1.13*0.25 ^c^ |
| Oligocheates | V = L*W^2^*530, Dry weight (µg) = V*1.13*0.25 ^c^ |
| Copepods | V = L*W^2^*560, Dry weight (µg) = V*1.13*0.25 ^c^ |

a: Chambers MR, Milne H (1978) Seasonal Variation in the Condition of some Intertidal Invertebrates of the Ythan Estuary, Scotland. Estuarine and Coastal Marine Science 8: 411-419.

b: Bolam SG (2004) Population structure and reproductive biology of Pygospio elegans (Polycheata: Spionidae) on an intertidal sandflat, Firth of Forth, Scotland. Invertebrate Biology 123: 260-268.

c: V is body volume in nl, L is length and W is maximum width in mm.

Feller RJ, Warwick RM (1988) Enegetics. In: Higgins RP, Thiel H, editors. Introduction to the Study of Meiofauna. London: Smithsonian Institution Press. pp. 181-196.

d: Omena EP, Amaeal ACZ (2001) Morphometric study of the nereidid Laeonereis acuta (Annelida: Polychaeta). Journal of the Marine Bioligical Association of the United Kingdom 81: 423-426.

e: W_10_ and L_10_ are width at the 10^th^ segment and length to 10^th^ segment, respectively.

Gillett DJ, Holland AF, Sanger DM (2005) Secondary production of a dominant oligocheate (Monopylephorus rubroniveus) in the tidal creeks of South Carolina and its ecosystem characteristics. Limnology and Oceanography 50: 566-577.

f: W is dry weight (µg), L is length (µm)

Tilbrook PJ, Block W (1972) Oxygen uptake in an Antarctic collembole Cryptopygus antarcticus. Oikos 23: 313-317.

g: W is weight CW is carapace width (Equation shows: combination of male and female)

Ozcan T, Bakir K, Katagan T (2009) Length/Width-Weight Relationships of the Mediterranean Green Crab Carcinus aestuarii Nardo, 1847 in the Homa Logoon Aegean Sea Turkey. Journal of Fishereis Science 3: 1-4.

h: Calculated for this study (n = 14; r^2^ = 0.998; p < 0.0001)

i: Leaper R (2000) Animal Body-Size Relationships: Patterns, Mechanisms and Implications. Ellon: The University of Aberdeen. pp. 121.

j: Crisp DJ (1971) Energy Flow Measurements. In: Holme NA, Mclntyre AD, editors. Methods for the study of Marine Benthos. First ed. Oxford and Edinburgh: Blackwell Scientific Publications. pp. 197-279.
